# Neurodegenerative disorders, metabolic icebergs, and mitohormesis

**DOI:** 10.1186/s40035-024-00435-8

**Published:** 2024-09-06

**Authors:** Matthew C. L. Phillips, Martin Picard

**Affiliations:** 1 Department of Neurology, Waikato Hospital, Hamilton, 3204 New Zealand; 2 Department of Medicine, University of Auckland, Auckland, 1142 New Zealand; 3 Division of Behavioral Medicine, Department of Psychiatry, Columbia University Irving Medical Center, New York, NY 10032 USA; 4 Department of Neurology, H. Houston Merritt Center, Columbia Translational Neuroscience Initiative, Columbia University Irving Medical Center, New York, NY 10032 USA; 5 New York State Psychiatric Institute, New York, NY 10032 USA; 6 Robert N Butler Columbia Aging Center, Columbia University Mailman School of Public Health, New York, NY USA

**Keywords:** Alzheimer’s disease, Parkinson’s disease, Amyotrophic lateral sclerosis, Huntington’s disease, Splitting, Lumping, Mitochondria, Mitotypes, Hormesis, Mitohormesis

## Abstract

Neurodegenerative disorders are typically “split” based on their hallmark clinical, anatomical, and pathological features, but they can also be “lumped” by a shared feature of impaired mitochondrial biology. This leads us to present a scientific framework that conceptualizes Alzheimer’s disease (AD), Parkinson’s disease (PD), amyotrophic lateral sclerosis (ALS), and Huntington’s disease (HD) as “metabolic icebergs” comprised of a tip, a bulk, and a base. The visible tip conveys the hallmark neurological symptoms, neurodegenerative regions, and neuronal protein aggregates for each disorder. The hidden bulk depicts impaired mitochondrial biology throughout the body, which is multifaceted and may be subdivided into impaired cellular metabolism, cell-specific mitotypes, and mitochondrial behaviours, functions, activities, and features. The underlying base encompasses environmental factors, especially modern industrial toxins, dietary lifestyles, and cognitive, physical, and psychosocial behaviours, but also accommodates genetic factors specific to familial forms of AD, PD, and ALS, as well as HD. Over years or decades, chronic exposure to a particular suite of environmental and genetic factors at the base elicits a trajectory of impaired mitochondrial biology that maximally impacts particular subsets of mitotypes in the bulk, which eventually surfaces as the hallmark features of a particular neurodegenerative disorder at the tip. We propose that impaired mitochondrial biology can be repaired and recalibrated by activating “mitohormesis”, which is optimally achieved using strategies that facilitate a balanced oscillation between mitochondrial stressor and recovery phases. Sustainably harnessing mitohormesis may constitute a potent preventative and therapeutic measure for people at risk of, or suffering with, neurodegenerative disorders.

## Background

*The cause is secret, but th’ effect is known.*

- *Ovid* [1].

Despite decades of investment and research, neurodegenerative disorders are becoming increasingly common. As of 2017, the global prevalence of Alzheimer’s disease (AD) and other dementias stood at 45 million people [2]. Another 8.5 million people were living with Parkinson’s disease (PD) [2]. Both AD and PD are doubling in prevalence every 20–30 years [3, 4]. Amyotrophic lateral sclerosis (ALS) afflicts 4.1–8.4 of every 100,000 people, or roughly 500,000 people globally [5]. The prevalence of ALS is increasing by approximately 70% every 25 years [6]. Lastly, Huntington’s disease (HD) afflicts 4.9 of every 100,000 people, or roughly 400,000 people globally [7]. The prevalence of HD increased by approximately 80% in the last 35 years. Although much of the rise in prevalence is explained by earlier diagnoses, population aging, and population growth, there may be a significant contribution from modifiable environmental factors [8, 9]. Unless we stem this rising tide, the ensuing socio-economic impact is poised to exceed the management capacity of many healthcare systems.

### Splitting and lumping

Rather than investing further resources into lines of research that have (so far) failed to stem the tide, it is worth considering how our chosen methods of classifying neurodegenerative disorders influence how we approach and treat them. In 1857, Darwin distinguished between two kinds of individuals, whom he called “splitters” and “lumpers” [10]. Splitters are people who make classifications based on distinct characteristics, which leads to multiple classification schemes that reflect these distinctions. Lumpers, by contrast, make classifications in a broad manner, which allows for ranges of characteristics to be classified into fewer entities. Ideally, a compromise between splitting and lumping might constitute the best approach to conceptualizing many of the medical disorders encountered in healthcare. However, due to a lengthy history of medical reductionism, the balance is currently heavily tilted towards splitting, which leads to difficulties when it comes to “putting the patient back together” [11].

**Fig. 1:**
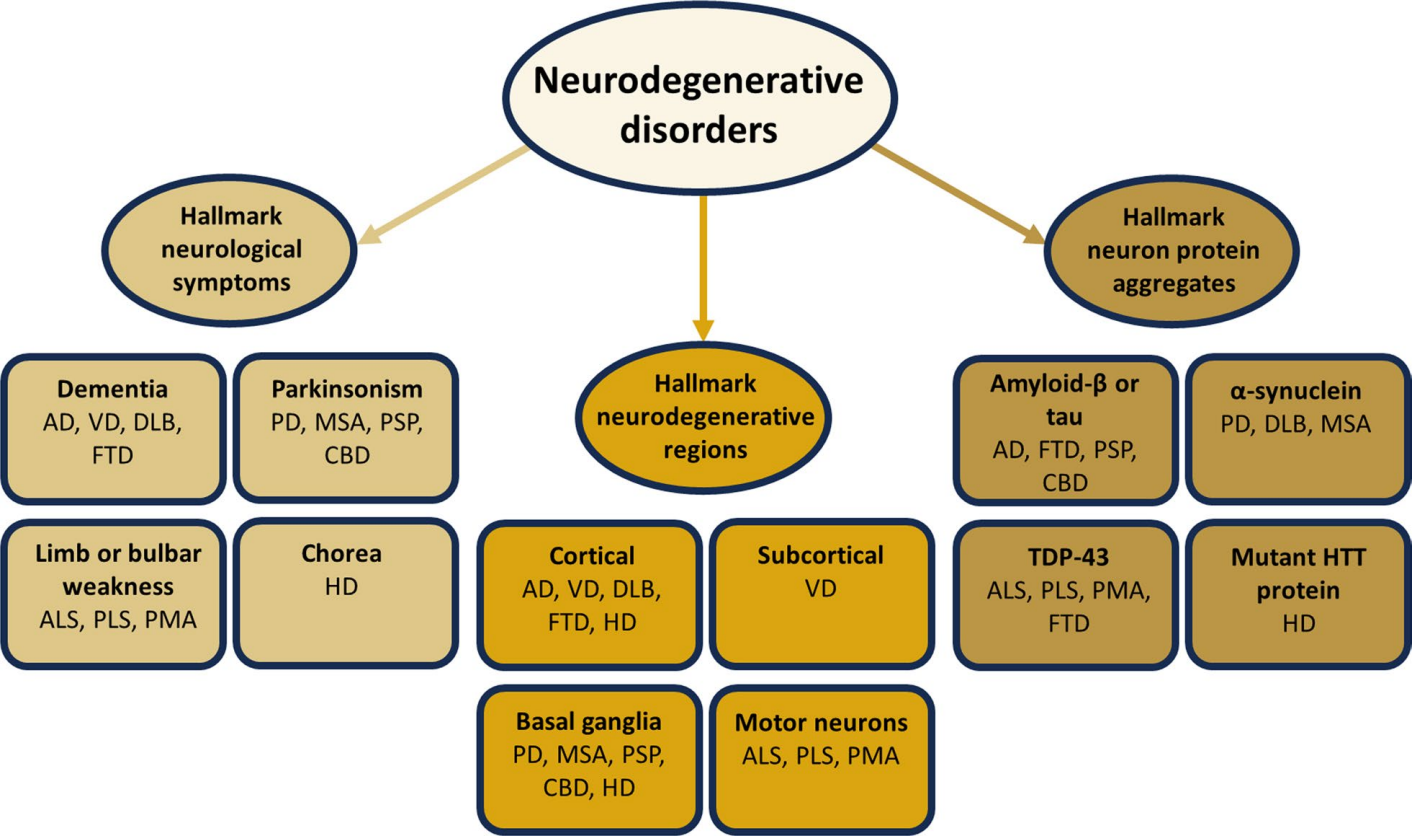
Splitting perspective of neurodegenerative disorders. *AD* Alzheimer’s disease, *VD* vascular dementia, *DLB* dementia with Lewy bodies, *FTD* frontotemporal dementia, *PD* Parkinson’s disease, *MSA* multiple systems atrophy, *PSP* progressive supranuclear palsy, *CBD* corticobasal degeneration, *ALS* amyotrophic lateral sclerosis, *PLS* primary lateral sclerosis, *PMA* progressive muscular atrophy, *HD* Huntington’s disease, *TDP-43* transactive response DNA binding protein 43, *HTT* Huntingtin

Neurodegenerative disorders are typically split based on their hallmark clinical, anatomical, and pathological features (Fig. 1) [12]. Splitting enables clinical diagnosis and management by identifying common clinico-pathological patterns, conveying prognostic information, and facilitating symptom-based treatments. However, it leads to several problems. First, splitting does not sufficiently emphasize the broader array of neurological symptoms in these disorders, particularly the non-cognitive symptoms of AD [13, 14], the non-motor symptoms of PD [15, 16], and the cognitive and behavioural symptoms of ALS and HD [17, 18]. Many of these symptoms arise from neurodegenerative changes outside the hallmark regions [14, 16, 19, 20]. Second, splitting does not explain why degenerative changes frequently occur in non-neurological tissues, particularly the skeletal muscles and heart [21–24]. We lack an understanding as to how these disorders manifest outside the nervous system. Third, splitting biases treatment efforts towards targeting and suppressing the hallmark symptoms and aggregates [25, 26]. However, these allopathic approaches have not produced clinically meaningful outcomes, and may lead to harm [27]. Essentially, splitting portrays neurodegenerative disorders as focal neurological disorders amenable to targeted, suppressive treatments, but fails to address their etiology and multisystemic nature.

**Fig. 2:**
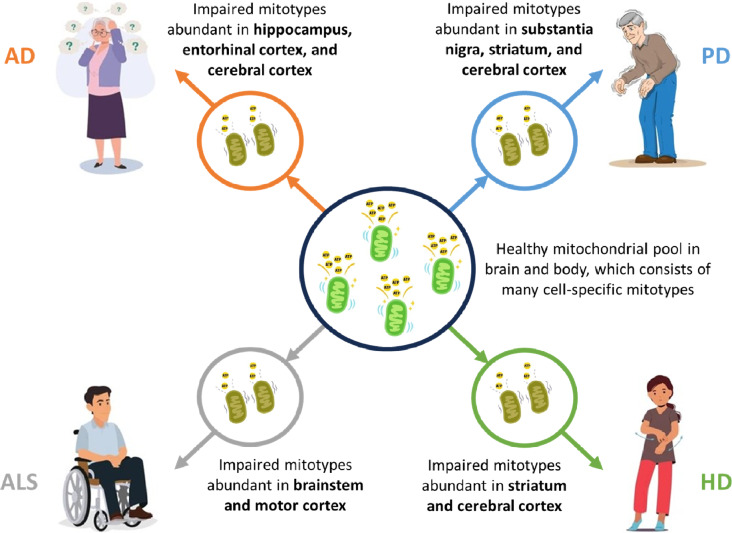
Lumping perspective of neurodegenerative disorders. *AD* Alzheimer’s disease, *PD* Parkinson’s disease, *ALS* amyotrophic lateral sclerosis, *HD* Huntington’s disease

Alternatively, this splitting paradigm can be integrated with a lumping perspective that emphasizes a shared feature of impaired mitochondrial biology, which has been documented across a wide range of neurodegenerative disorders, including AD, PD, ALS, and HD (Fig. 2) [28–30]. Although traditionally described as cell “powerhouses”, mitochondria are more comprehensively described as cell “processors” that coordinate energy and metabolism throughout the body [31, 32]. Distributed mitochondrial networks sense and communicate bioenergetic states to ensure that cellular behaviours match energy availability and demands [32]. Mitochondria also coordinate an array of processes, including epigenetic modifications, adenosine triphosphate (ATP) production, reactive oxygen species (ROS) emission, hormone biosynthesis, and neurotransmitter metabolism [31–34]. Moreover, in the same way that organisms are composed of highly specialized organs and cell types that perform complementary functions, recent evidence has revealed a diverse family of cell-specific mitochondrial phenotypes or “mitotypes” throughout the brain, each of which is energetically and metabolically optimized to meet the requirements of a particular subset of neurons and brain regions [35–38]. Based on this emerging understanding of mitochondria, a lumping perspective recognizes the hallmark clinical, anatomical, and pathological features of each neurodegenerative disorder as offshoots of a common bioenergetic and metabolic etiology, which maximally impacts subsets of mitotypes abundant in the afflicted brain regions. Lumping explains the diversity of symptoms and degenerative changes documented in other areas of the nervous system, the skeletal muscles, and heart, as all these tissues are rich in mitochondria. It also implies that approaches geared towards allopathic targeting and suppression are of limited benefit in the setting of impaired mitochondrial biology, which fundamentally requires a restorative approach [9, 39]. Essentially, lumping neurodegenerative disorders by their impaired mitochondrial biology enables them to be conceptualized as multisystemic disorders in need of multisystemic, restorative therapies.

### Perspective

We will discuss how the current splitting paradigm can be integrated with a lumping perspective, which converges upon impaired mitochondrial biology as the etiology and core disease in the most common neurodegenerative disorders. This integration will expand upon previous similar proposals by providing a scientific framework that portrays how this common (lumped) disease process drives the hallmark (split) features of each disorder. This framework will then be utilized to identify and discuss preventative and therapeutic strategies that show promise in repairing and recalibrating mitochondrial biology. Supportive evidence will be mostly derived from human studies, emphasizing interventional studies where possible.

## Neurodegenerative disorders as metabolic icebergs

**Fig. 3:**
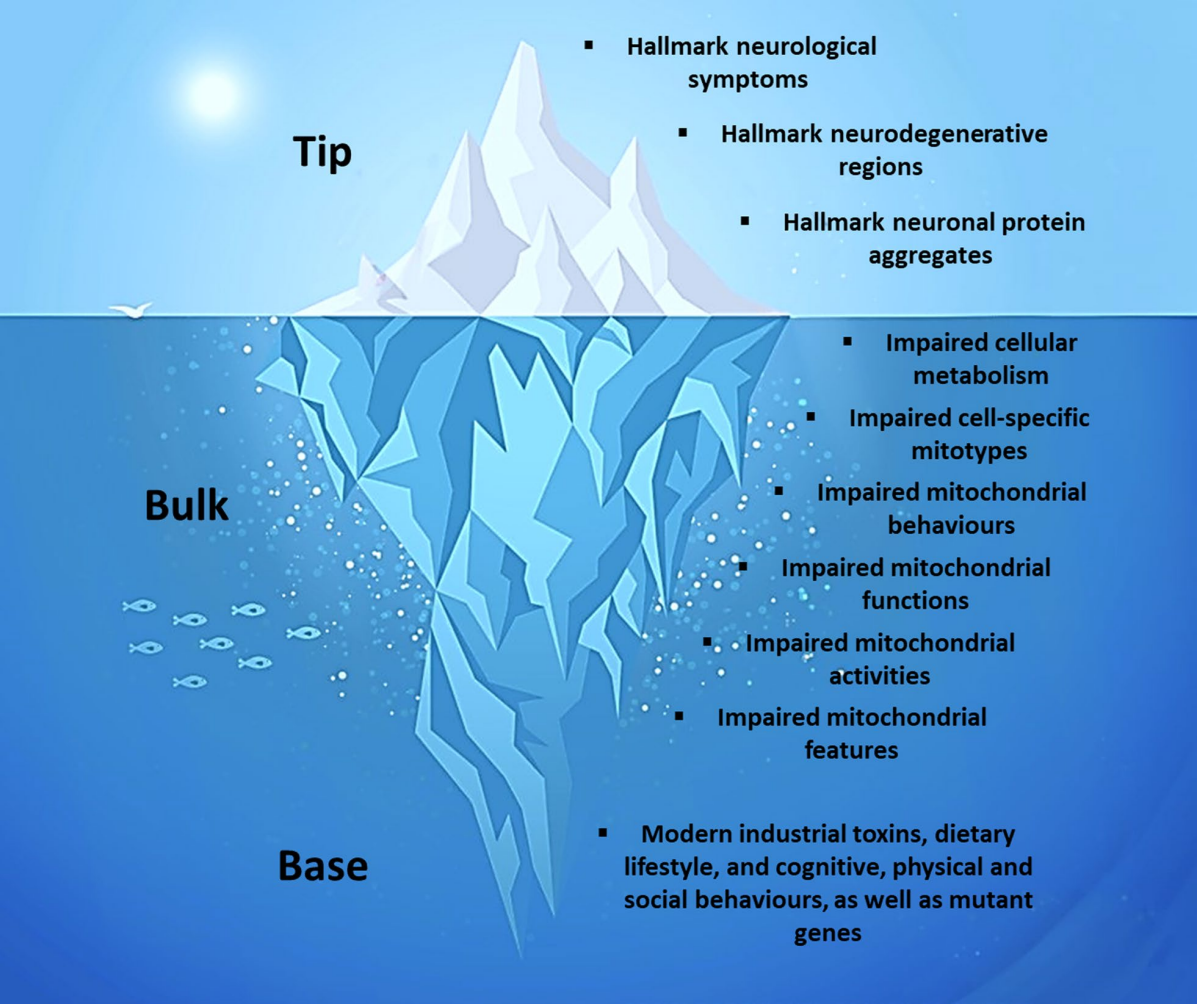
The metabolic iceberg view of neurodegenerative disorders. This framework conveys the pathogenesis of each neurodegenerative disorder, which commences at the base, proceeds through the bulk, and eventually surfaces at the tip

Each neurodegenerative disorder may be conceptualized as a “metabolic iceberg” comprised of a tip, a bulk, and a base (Fig. 3). The hallmark clinical, anatomical, and pathological features are illustrated in the tip, which emerge as downstream effects of the core disease, impaired mitochondrial biology, in the bulk, which in turn is triggered by a particular suite of environmental and genetic factors in the base. This section will outline a comprehensive scientific framework for conceptualizing the pathogenesis of any neurodegenerative disorder. Subsequent sections will apply this framework to the common neurodegenerative disorders that plague modern healthcare, specifically AD, PD, ALS, and HD.

### Tip of the iceberg

The tip of the metabolic iceberg conveys the clinical, anatomical, and pathological features for each neurodegenerative disorder, the most visible of which are the “hallmark” features. The highest and most visible level, the level of the organism, encapsulates the *hallmark neurological symptoms* for each disorder, which are diagnosed and managed by clinicians. The middle level, the level of body systems, organs, and tissues, describes the *hallmark neurodegenerative regions*, which may be identified on neuroimaging and occasionally addressed with surgical techniques [40, 41]. The lowest level, the level of the cell, comprises the *hallmark neuronal protein aggregates*, which may be identified by circulating biomarkers and are typically addressed with targeted, suppressive approaches [25, 26].

### Bulk of the iceberg

The bulk of the metabolic iceberg represents impaired mitochondrial biology throughout the body, which lies below the clinical waterline and remains relatively unexplored. Impaired mitochondrial biology may be subdivided into several levels, with several facets attributed to each [31]. The highest and most complex level encapsulates *impaired cellular metabolism*, which maximally impacts neurons in the hallmark neurodegenerative regions [42–45], but also affects other metabolically active non-neuronal cells throughout the brain and body, such as glia and myocytes [46–48]. The second level describes *impaired cell-specific mitotypes* (characteristics of mitochondria relevant to a specific cell type), such as an altered mitochondrial content and distribution within neurons. The third level comprises *impaired mitochondrial behaviours* (goal-directed processes involving the whole mitochondrion), which include disruptions in mitochondrial fusion and fission, motility, biogenesis (mitogenesis), and autophagy (mitophagy). The fourth level describes *impaired mitochondrial functions* (integrated processes involving multiple mitochondrial components), such as decreased ATP synthesis, increased ROS emission, dysregulated calcium regulation, and altered steroidogenesis. The fifth level comprises *impaired mitochondrial activities* (processes involving individual mitochondrial components), which include reduced enzymatic activities in the tricarboxylic acid (TCA) cycle and electron transport chain (ETC). The lowest level denotes *impaired mitochondrial features* (static molecular components), such as altered mitochondrial shape and size, as well as damage to cristae and mitochondrial DNA (mtDNA).

### Base of the iceberg

The base of the metabolic iceberg is represented by environmental and genetic factors that damage mitochondria or force maladaptive adjustments in their biology. Observational evidence in humans implicates environmental factors related to modern lifestyles as the key drivers of impaired mitochondrial biology, particularly in sporadic AD, PD, and ALS [8, 9]. First, substantial evidence implicates *modern industrial toxins*, such as heavy metals, air pollutants, pesticides, and chemicals, as major contributory factors to the neurodegenerative process [8]. These toxins accumulate in mitochondria and induce oxidative damage [49]. Second, growing evidence implicates the *modern dietary lifestyle*, which is characterized by a high intake of processed, carbohydrate-rich foods combined with multiple daily meals and snacks [9]. This dietary lifestyle is evolutionarily unprecedented and leads to a combination of chronic energy overload and insufficient recovery time that drives oxidative damage in mitochondria [50]. Third, *modern cognitive, physical, and psychosocial behaviours* lead to deficiencies in authentic cognitive, physical, and psychosocial interactions, as well as disrupted sleep and rest states. These behavioural factors get “under the skin” through an array of psychobiological pathways that energetically converge upon mitochondria [51]. Interventional evidence derived from human, animal, and cell models also demonstrates that an array of genetic factors can drive impaired mitochondrial biology and trigger familial forms of AD, PD, and ALS, as well as HD [52–55]. Most of these are gene mutations, which express toxic protein products that directly damage mitochondria. However, a loss of normal protein function can also compromise mitochondria by forcing maladaptive adjustments in their biology.

### Iceberg pathogenesis

The metabolic iceberg framework conveys a comprehensive overview of the pathogenesis of each neurodegenerative disorder. This process commences at the base, proceeds through the bulk, and eventually surfaces at the tip. Based on this framework, a detailed understanding of the tip enables clinical diagnosis and management, but it provides limited tractable information to guide prevention or therapy. Conversely, an understanding of the bulk and the base sheds light on strategies that may act in the interest of prevention and therapy by inducing “salugenesis” [39].

Neurodegenerative disorders commence at the base. Chronic exposure to a particular “suite” of environmental and genetic factors damages mitochondria or forces maladaptive adjustments, which elicits a particular trajectory of impaired mitochondrial biology that eventually surfaces with the hallmark features of AD, PD, ALS, or HD. Under the metabolic iceberg framework, sporadic AD, PD, and ALS are primarily driven by environmental factors. Many of these factors are broadly implicated in the etiology of multiple disorders, such as lead exposure in AD, PD, and ALS [56–58]. Others are more specifically linked to the etiology of a particular disorder, such as air particulate matter exposure in AD [56], rotenone exposure in PD [57], and electrical exposure in ALS [58]. Familial forms of AD, PD, and ALS, and all cases of HD, are primarily driven by genetic factors that impair mitochondrial biology. Most of these are gene mutations, which act as highly specific triggers of a particular disorder [52–55].

Neurodegenerative disorders proceed through the bulk for years or decades. During this time, the suite of instigating factors induces multifaceted, multisystemic impairments in mitochondrial biology that maximally impact subsets of mitotypes abundant in the eventual hallmark neurodegenerative regions particular to AD, PD, ALS, or HD. Mitochondria in these regions begin to exhibit impaired biology on multiple levels, including decreased ATP production and increased ROS emission leading to oxidative damage [59], which may represent the earliest event in pathogenesis [60]. Given that mitochondria constitute the main intracellular source of ROS [61], mitochondrial oxidative damage elicits an upsurge in ROS emission, which may be followed by a vicious cycle of ROS-induced ROS release that drives further oxidative damage [62]. The ROS overflow also triggers neuroinflammation and lysosome dysfunction, both of which can, in turn, induce further mitochondrial damage [63–65]. Depending on the particular suite of initiating environmental and genetic factors, the trajectory of impaired mitochondrial biology maximally impacts subsets of mitotypes abundant in the hallmark neurodegenerative regions particular to AD, PD, ALS, or HD, which leads to a relatively pronounced failure of energy and metabolism in these regions. This is consistent with findings from positron emission tomography (PET) studies showing that deficits in mitochondrial energy metabolism occur early in the hallmark regions, often years before the clinical symptoms [42–45].

The hallmark features of AD, PD, ALS, or HD surface at the tip. These features represent a late-stage effect that follows years or decades of impaired mitochondrial biology in the hallmark neurodegenerative regions. Perhaps controversially, the metabolic iceberg largely positions the hallmark neuronal protein aggregates as downstream consequences of impaired mitochondrial biology. This conceptualization is supported by studies demonstrating that mitochondria-mediated oxidative stress both precedes and promotes the deposition of the aggregates [66–71]. However, the iceberg framework can also accommodate evidence that the aggregates can, in turn, damage mitochondria in a reciprocal manner [72]. Beyond the hallmark regions, mitochondria in other areas of the nervous system are impaired, which leads to the broader array of neurological symptoms observed in these disorders, including the non-cognitive symptoms of AD [13, 14], the non-motor symptoms of PD [15, 16], and the cognitive and behavioural symptoms of ALS and HD [17, 18]. Impaired mitochondrial biology outside the nervous system also leads to degenerative changes in the skeletal muscles, heart, and other metabolically active tissues, followed by more generalized symptoms such as muscle wasting and weight loss [73].

## AD as a metabolic iceberg

**Fig. 4:**
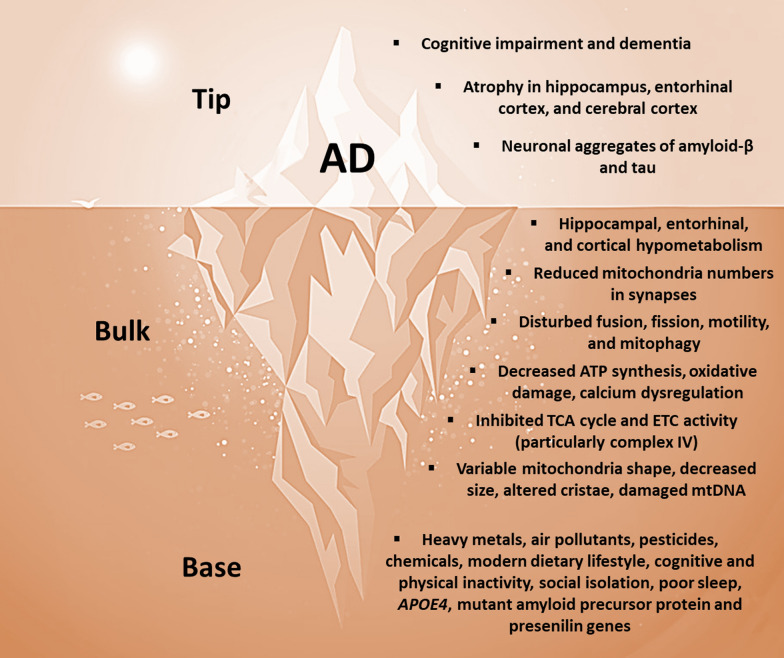
AD as a metabolic iceberg. *AD* Alzheimer’s disease, *ATP* adenosine triphosphate, *TCA* tricarboxylic acid, *ETC* electron transport chain, *mtDNA* mitochondrial DNA, *APOE4* apolipoprotein E4

AD may be conceptualized as a metabolic iceberg, with the hallmark clinical, anatomical, and pathological features emerging as late-stage effects of impaired mitochondrial biology (Fig. 4). We propose that chronic exposure to a particular suite of environmental and genetic factors elicits a trajectory of impaired mitochondrial biology that maximally impacts subsets of mitotypes abundant in the hippocampus, entorhinal cortex, and cerebral cortex, which eventually surfaces as the hallmark features of AD.

### AD: base of the iceberg

Human observational studies implicate environmental factors related to modern lifestyles as primary drivers of impaired mitochondrial biology in AD [8, 9]. Modern industrial toxins include heavy metals (such as aluminum, lead, copper, and arsenic), air pollutants (such as particulate matter and ozone), pesticides (such as organochlorines and organophosphates), flame retardants, and plasticizers [56]. The modern dietary lifestyle is the primary driver for the rising rates of mid-life obesity, type 2 diabetes, and hypertension, each of which increases the risk of developing AD [74]. Alcohol is an established risk factor [75]. Fewer years of formal education, physical inactivity, depression, chronic social isolation, and sleep disturbances are also associated with an increased risk of developing AD [74, 75]. Beyond these environmental factors, a number of genetic factors link impaired mitochondrial biology to the pathogenesis of AD [52]. Apolipoprotein E4 (*APOE4*), a major genetic risk factor for AD, targets and disrupts mitochondrial ATP production and ROS emission [76]. The mutant amyloid precursor protein accumulates in the protein import channels of mitochondria, where it inhibits complex IV activity and increases ROS emission [77]. Mutant presenilins 1 and 2 disrupt mitochondrial calcium signalling, which leads to decreased ATP production, increased ROS emission, and cell death [78].

### AD: bulk of the iceberg

Human neuroimaging and post-mortem studies show that impaired mitochondrial biology occurs early in the pathogenesis of AD [79]. PET studies have detected glucose hypometabolism in the hippocampus, entorhinal cortex, and cerebral cortex, which may occur 30–40 years prior to the clinical symptoms [42]. Cerebral hypometabolism develops in concert with impaired brain insulin signalling, which has led to AD being described as “type 3 diabetes” [80]. Phenotypically, cortical neuron mitochondria show reduced numbers, particularly within synapses [81, 82]. They also display a disturbed balance between fusion and fission, disrupted motility, and defective mitophagy [83–85]. Functionally, cortical neuron mitochondria exhibit decreased ATP synthesis and oxidative damage, which not only precede the clinical symptoms [86], but can also precede and promote the deposition of amyloid-β and tau [66, 67]. They also show evidence of calcium dysregulation [87]. Moreover, cortical neuron mitochondria demonstrate inhibited enzyme activities in the TCA cycle and ETC (particularly complex IV) [88]. Lastly, hippocampal and cortical neuron mitochondria display increased variability in shape, decreased size, altered cristae, and oxidative damage to mtDNA [89].

### AD: tip of the iceberg

After years or decades of impaired mitochondrial biology, the tip emerges as AD, which is typically diagnosed in the setting of progressive cognitive impairment culminating in dementia [90]. This clinical progression may be accompanied by neurodegenerative changes in the hippocampus, entorhinal cortex, and cerebral cortex, as well as biomarker evidence of amyloid-β and tau [91]. However, numerous non-cognitive symptoms may accompany the hallmark cognitive symptoms, which include loss of smell, pain syndromes, gait dysfunction, agitation, aggression, circadian rhythm disruptions, sleep disturbances, and weight loss [13, 14]. Accordingly, neurodegenerative changes are present in other areas of the nervous system, such as the olfactory bulb and cervical spine [13, 14], and amyloid-β and tau aggregates may be detected in virtually all tissues outside the nervous system, including the skeletal muscles, heart, adrenal glands, kidneys, liver, pancreas, stomach, bowel, spleen, lymph nodes, thyroid, aorta, lung, testes, and ovaries [21].

## PD as a metabolic iceberg

**Fig. 5:**
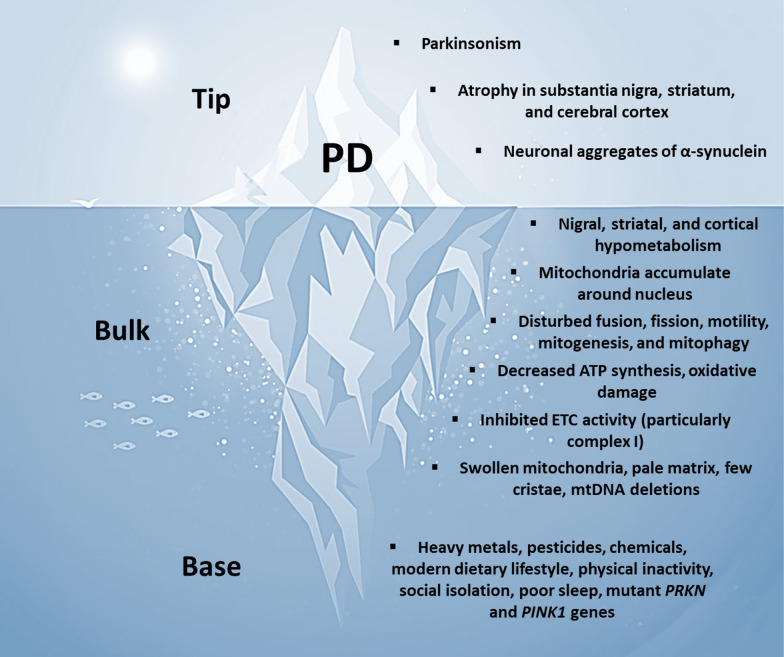
PD as a metabolic iceberg. *PD* Parkinson’s disease, *ATP* adenosine triphosphate, *ETC* electron transport chain, *mtDNA* mitochondrial DNA, *PRKN* Parkin, *PINK1* phosphate and tensin homolog-induced kinase 1

PD may also be conceptualized as a metabolic iceberg, with the hallmark clinical, anatomical, and pathological features emerging as late-stage effects of impaired mitochondrial biology (Fig. 5). We propose that chronic exposure to a particular suite of environmental and genetic factors elicits a trajectory of impaired mitochondrial biology that maximally impacts subsets of mitotypes abundant in the substantia nigra, striatum, and cerebral cortex, which eventually surfaces as the hallmark features of PD.

### PD: base of the iceberg

Human observational studies implicate environmental factors related to modern lifestyles as primary drivers of impaired mitochondrial biology in PD [8, 9]. Modern industrial toxins include heavy metals (such as iron, lead, manganese, and mercury), pesticides (such as organophosphates, rotenone, and paraquat), and solvents [57]. Notably, rotenone is a mitochondrial complex I inhibitor that induces parkinsonism in animal models [92]. Another mitochondrial toxin and complex I inhibitor, 1-methyl-4-phenyl-1,2,3,6-tetrahydropyridine, triggers parkinsonism in humans [93]. The modern dietary lifestyle is associated with an increased risk of PD progression—foods typically low in carbohydrates (or, when carbohydrates are present, they are high in fiber) are associated with reduced progression, whereas foods associated with more rapid progression are typically high in processed carbohydrates [94]. Lower coffee consumption is an established risk factor [95]. Physical inactivity and social withdrawal are also associated with an increased risk of PD development and progression [95, 96]. Beyond these environmental factors, a number of genetic factors link impaired mitochondrial biology to the pathogenesis of PD [53]. Mutations in parkin (*PRKN*) or phosphate and tensin homolog-induced kinase 1 (*PINK1*) prevent cells from responding to mitochondrial damage by altering the balance of fusion to fission and disrupting mitophagy, which leads to familial forms of PD [97, 98].

### PD: bulk of the iceberg

Human neuroimaging and post-mortem studies show that impaired mitochondrial biology occurs early in the pathogenesis of PD [99]. PET studies have detected glucose hypometabolism in the substantia nigra, striatum, and cerebral cortex [43]. Phenotypically, platelet mitochondria exhibit an altered distribution, preferentially accumulating around the nucleus [100]. Neurons also show decreased levels of peroxisome proliferator-activated receptor-γ coactivator-1α (PGC-1α), a key regulator of mitogenesis [101], and mitochondria display disturbances in fusion and fission, motility, and mitophagy in familial forms of PD [53]. Functionally, synaptic mitochondria are extrapolated to have a 35%–40% decrease in ATP synthesis based on the reported decrease in complex I activity [102], as well as elevated markers of oxidative damage [103]. This mitochondria-mediated oxidative stress precedes and promotes the deposition of α-synuclein [68, 69]. Moreover, mitochondria from substantia nigra neurons, skeletal myocytes, and platelets show inhibited enzyme activities in the ETC (particularly complex I) [104–106]. Lastly, platelet-derived mitochondria from PD cybrid cells exhibit abnormal features, which include an enlarged or swollen shape, pale matrix, and few remaining cristae [107], and substantia nigra neuron mitochondria in older people with parkinsonism display abundant deletions in mtDNA [108].

### PD: tip of the iceberg

After years or decades of impaired mitochondrial biology, the tip emerges as PD, which is typically diagnosed in the setting of motor symptoms consistent with parkinsonism [109]. These symptoms may be accompanied by neurodegenerative changes in the substantia nigra and striatum, as well as biomarker evidence of oligomeric α-synuclein [110]. However, numerous non-motor symptoms may accompany the hallmark motor symptoms, which include loss of smell, pain syndromes, depression, anxiety, urinary and gastrointestinal dysfunction, sleep disorders, cognitive impairment, apathy, and weight loss [15, 16]. Accordingly, neurodegenerative changes are present in other areas of the nervous system, such as the peripheral, autonomic, and enteric nervous systems [15, 16], and α-synuclein aggregates may be detected outside the nervous system, including in the digestive tract, skeletal muscles, heart, adrenal glands, kidneys, urogenital system, and skin [22].

## ALS as a metabolic iceberg

**Fig. 6:**
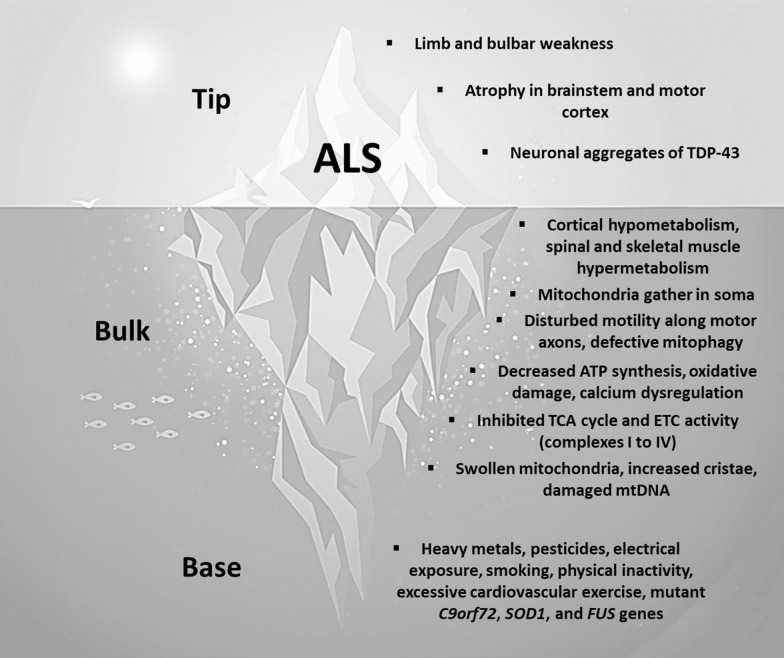
ALS as a metabolic iceberg. *ALS* amyotrophic lateral sclerosis, *TDP-43* transactive response DNA binding protein 43, *ATP* adenosine triphosphate, *TCA* tricarboxylic acid, *ETC* electron transport chain, *mtDNA* mitochondrial DNA, *C9orf72* chromosome 9 open reading frame 72, *SOD1* superoxide dismutase 1, *FUS* fused in sarcoma

ALS may also be conceptualized as a metabolic iceberg, with the hallmark clinical, anatomical, and pathological features emerging as late-stage effects of impaired mitochondrial biology (Fig. 6). We propose that chronic exposure to a particular suite of environmental and genetic factors elicits a trajectory of impaired mitochondrial biology that maximally impacts subsets of mitotypes abundant in the brainstem and motor cortex, which eventually surfaces as the hallmark features of ALS.

### ALS: base of the iceberg

Human observational studies implicate environmental factors related to modern lifestyles as primary drivers of impaired mitochondrial biology in ALS [8, 9]. Modern industrial toxins include heavy metals (such as lead, iron, manganese, and selenium) and pesticides (such as organochlorines, pyrethoids, herbicides, and fumigants) [58]. Electrical occupations involving repeated exposures to electrical shocks or electromagnetic fields are associated with an increased risk of developing ALS [111]. Smoking is an established risk factor [112]. Exercise may be a two-edged sword, as people engaged in organized sport have a 51% lower risk of developing ALS, but professional athletes have a 59% higher risk [113]. Higher cardiovascular fitness, but not muscle strength, is also associated with an increased risk of ALS later in life [114]. Beyond these environmental factors, a number of genetic factors link impaired mitochondrial biology to the pathogenesis of ALS [54]. Mutations in chromosome 9 open reading frame 72 (*C9orf72*) compromise mitochondrial function and increase oxidative stress in motor neurons [115]. Superoxide dismutase 1 (SOD1) normally breaks down superoxide; mutations in *SOD1* can induce oxidative damage in motor neuron mitochondria and trigger the clinical symptoms of ALS [116]. Mutant fused in sarcoma (*FUS*), which also promotes mitochondrial damage [117], can trigger aggressive familial forms of ALS [118].

### ALS: bulk of the iceberg

Human neuroimaging and post-mortem studies show that impaired mitochondrial biology occurs early in the pathogenesis of ALS [54]. PET studies have detected glucose hypometabolism in the frontal, motor, and occipital cortices [44]. Phenotypically, motor neuron mitochondria display an altered distribution, preferentially gathering in the soma [119]. Disturbed mitochondrial transport along motor neuron axons and defective mitophagy are among the earliest pathophysiological events [120]. Functionally, spinal cord neurons, lymphocytes, and skeletal myocytes exhibit decreased ATP synthesis, oxidative damage, and calcium dysregulation [121–123]. Mitochondria-mediated oxidative stress can both precede and promote the deposition of transactive response DNA binding protein 43 (TDP-43) [70, 71]. Mitochondria in spinal cord neurons, lymphocytes, and skeletal myocytes also demonstrate inhibited activities in citrate synthase and ETC complexes I–IV [121, 122, 124, 125]. Moreover, motor neuron mitochondria show abnormal features, including a swollen appearance, markedly increased cristae, and oxidative damage to mtDNA [119, 121]. Altogether, these multifaceted mitochondrial alterations would be expected to lead to a series of energetically costly adjustments, including compensatory hypermetabolism in the spinal cord and skeletal muscles [126, 127]. Despite these adjustments, impaired mitochondrial biology still ripples out at the level of the organism.

### ALS: tip of the iceberg

After years or decades of impaired mitochondrial biology, the tip emerges as ALS, which is typically diagnosed in the setting of limb or bulbar weakness supported by characteristic changes on electromyography [128]. These symptoms may be accompanied by neurodegenerative changes in the brainstem and motor cortex, as well as biomarker evidence of TDP-43 [129, 130]. However, many additional symptoms may accompany the hallmark limb or bulbar weakness, which include executive dysfunction, language impairment, disinhibition, loss of empathy, apathy, muscle wasting, and weight loss [17]. Accordingly, neurodegenerative changes are present elsewhere in the nervous system, such as other regions of cerebral cortex, the hippocampus, and cerebellum [19]. Outside the nervous system, TDP-43 aggregates may be detected in the skeletal muscles and heart [23].

## HD as a metabolic iceberg

**Fig. 7:**
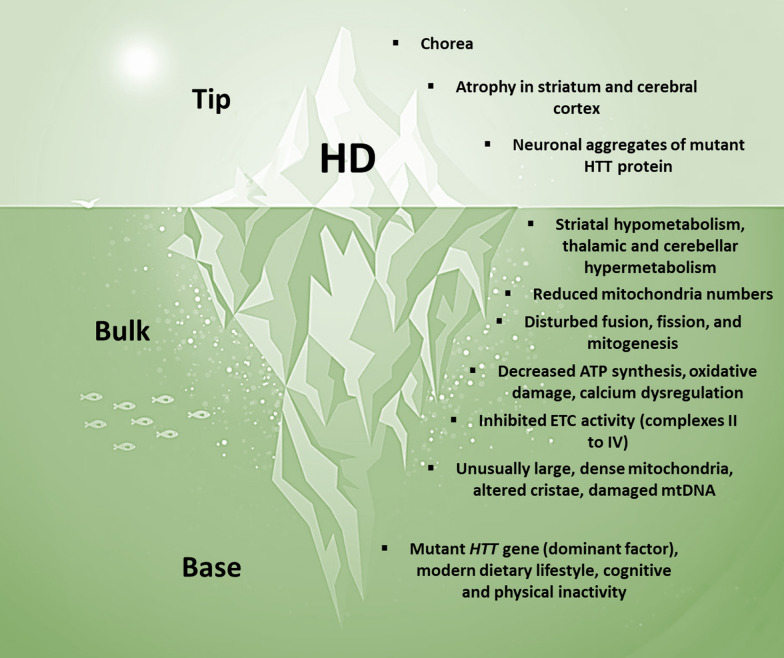
HD as a metabolic iceberg. *HD* Huntington’s disease, *HTT* Huntingtin, *ATP* adenosine triphosphate, *ETC* electron transport chain, *mtDNA* mitochondrial DNA

Although HD is commonly described as a monogenic disorder, it can still be conceptualized as a metabolic iceberg, with the hallmark clinical, anatomical, and pathological features emerging as late-stage effects of impaired mitochondrial biology (Fig. 7). We propose that chronic exposure to mutant huntingtin (HTT) elicits a trajectory of impaired mitochondrial biology that maximally impacts subsets of mitotypes abundant in the striatum and cerebral cortex, which eventually surfaces as the hallmark features of HD.

### HD: base of the iceberg

The *HTT* gene codes for the HTT protein, which is ubiquitously expressed throughout the body [131]. This protein is involved in many different processes related to immunity, gene expression, cellular metabolism, and a range of mitochondrial functions and activities [55, 132, 133]. Beyond a certain threshold, a CAG repeat expansion in the *HTT* gene leads to the expression of the mutant HTT protein, which can facilitate mitochondrial damage on many levels [55]. Moreover, since normal HTT protein is essential for mitochondrial bioenergetics and metabolism [134], a loss of normal function would also be expected to trigger maladaptive adjustments in mitochondrial biology [135]. Despite the pivotal role of mutant HTT in triggering and driving HD, growing evidence indicates that its symptomatic expression is significantly influenced by environmental factors [136, 137], such that even monozygotic twins bearing the same number of CAG repeats may show considerable differences in the age of onset and nature of their HD symptoms [138, 139]. Malonate, a mitochondrial toxin and complex II inhibitor, can trigger striatal lesions in animal models that closely resemble those of HD [140]. Dietary lifestyle may influence symptom onset, since asymptomatic mutation carriers with a higher calorie and dairy intake develop HD symptoms at an earlier age [141]. Cognitive and physical lifestyle factors may also be influential, given that sedentary behaviour is associated with an earlier onset and greater severity of symptoms [142].

### HD: bulk of the iceberg

Human neuroimaging and post-mortem studies show that impaired mitochondrial biology occurs early in the pathogenesis of HD [55]. PET studies have detected glucose hypometabolism in the striatum, which often occurs years before the clinical symptoms [45]. Phenotypically, striatal neuron mitochondria exhibit reduced numbers [143]. Striatal and cortical neuron mitochondria display a loss of balance between fusion and fission [143, 144], and striatal neurons in asymptomatic mutation carriers show decreased expression of the key mitogenesis regulator PGC-1α [145]. Functionally, ATP synthesis is decreased in the skeletal muscle of both asymptomatic and symptomatic mutation carriers [146]. There is also evidence of increased oxidative damage in the cerebral cortex [144], as well as calcium dysregulation in lymphoblasts [147]. Striatal neuron mitochondria demonstrate decreased enzyme activities in ETC complexes II–IV [148], which is accompanied by inhibited complex I activity in skeletal myocytes [149]. Moreover, cortical and skeletal muscle mitochondria exhibit abnormal features, such as unusually large, dense mitochondria with altered cristae and oxidative damage to their mtDNA [149–151]. Altogether, these multifaceted mitochondrial alterations would be expected to lead to a series of energetically costly adjustments, including compensatory hypermetabolism in the thalamus and cerebellum [152]. Despite these adjustments, impaired mitochondrial biology still ripples out at the level of the organism.

### HD: tip of the iceberg

After years or decades of impaired mitochondrial biology, the tip emerges as HD, which is typically diagnosed in the setting of involuntary hyperkinetic movements (chorea) supported by confirmatory genetic testing [153]. These symptoms may be accompanied by neurodegenerative changes in the striatum and cerebral cortex, as well as biomarker evidence of the mutant HTT protein [154, 155]. However, many additional symptoms may accompany the hallmark choreiform symptoms, which include poor social cognition, irritability, depression, anxiety, apathy, psychosis, skeletal muscle wasting, weight loss, and heart failure [18]. Accordingly, neurodegenerative changes are present elsewhere in the nervous system, such as the thalamus, cerebellum, and brainstem [20]. Outside the nervous system, skeletal and cardiac myocytes show aberrations related to the expression of the mutant HTT protein [24].

## Mitohormesis

In 1932, a biphasic response to harmful substances was described, called “hormesis” [156]. Hormesis captures the observation that high concentrations of harmful substances lead to cell damage, whereas low levels induce adaptive responses that improve the body’s defence mechanisms and resilience [157]. Hormesis is particularly applicable to the generation of ROS, which drive cellular damage and aging at high levels [158, 159], but at low levels stimulate an adaptive mitochondrial stress response that ultimately enhances lifespan [160–162]. Given that mitochondria generate the majority of intracellular ROS, the hormesis concept was later recharacterized as “mitohormesis”, which highlights mitochondria as central coordinators of hormesis [163]. Mitohormesis encapsulates the idea that exposing mitochondria to a challenging (but not excessive) stressor leads to a recalibration of mitochondrial biology that subsequently protects the organelles against higher, normally harmful exposures to similar stressors in the future [164, 165]. Common stressors linked to mitohormesis include environmental toxins, dietary factors, cognitive stimulation, physical exercise, extreme temperatures, and hypoxia [157, 166–168]. Most of human evolution has been characterized by a mitohormesis-activating lifestyle [169], whereas many aspects of the modern lifestyle compromise mitohormesis.

We propose that mitohormesis may be optimally activated by strategies that facilitate a balanced oscillation between challenging (but not excessive) stressor phases, which temporarily disrupt mitochondrial biology, and complete (but not excessive) recovery phases, which provide adequate time for mitochondria to repair and recalibrate prior to the next stressor (Table 1). Previous similar proposals have focused on the benefits of the stressor phase (such as diet and exercise) in activating mitohormesis, with little or no emphasis on the crucial role of the recovery phase in optimizing it. The perspective presented here expands the mitohormesis concept by emphasizing that it is optimally activated by a *balanced oscillation* between mitochondrial stressor and recovery phases, rather than relying on one phase over the other. By manipulating these phases to achieve an optimal balance, it may be possible to sustainably harness mitohormesis as a preventative and therapeutic measure for people at risk of, or suffering with, neurodegenerative disorders.

**Table 1:** Strategies that activate mitohormesis, which is optimally activated by a *balanced oscillation* between mitochondrial stressor and recovery phases

Lifestyle factors	Stressor phase	Recovery phase
Industrial toxins	*Exposure* Transient, low-dose exposures to heavy metals, air pollutants, pesticides, and chemicals	*Non-exposure* Prolonged, near-zero dose exposures to heavy metals, air pollutants, pesticides, and chemicals
Dietary lifestyle	*Feeding* Evolutionary hunter-gatherer diets (minimally processed, carbohydrate-restricted)	*Fasting* Fasting protocols (particularly intermittent fasting, coffee permitted)
Cognitive behaviours	*Cognitive stimulation* Cognitive training, activities that demand sustained attention and promote engagement	*Sleep* Adequate sleep opportunity in a dedicated environment, sleep hygiene measures
Physical behaviours	*Exercise* Relatively intensive, strength-based exercise sessions	*Deep rest* Contemplative practices (such as breathwork, mindfulness, and meditation), prioritizing sufficient rest and recovery from exercise and psychosocial engagement
Psychosocial behaviours	*Psychosocial engagement* Social activities, networks, supports, and relationships	

### Industrial toxins

Over the last 250 years of the industrial age, humans have been increasingly exposed to a variety of heavy metals, air pollutants, pesticides, and chemicals [8]. In evolution, multicellular organisms were frequently exposed to selective pressure from toxic elements such as oxygen, iron, copper, manganese, and zinc [170]. However, these toxic exposures occurred over millions of years, which provided ample time for primitive organisms to evolve protective mechanisms. By contrast, the relatively abrupt human exposure to industry-derived toxins has not provided adequate evolutionary time for adaptation to occur. Many industrial toxins selectively target and elicit oxidative damage in mitochondria [49], which are vulnerable owing to a relative deficiency in DNA repair mechanisms [171]. Of particular concern are mitotoxicants with long half-lives, such as heavy metals and air particulate matter, which may not be removed from mitochondria [172]. Toxin-mediated mitochondrial damage facilitates an upsurge in ROS emission, which leads to a vicious cycle of ROS-induced ROS release and further damage [62]. Simultaneously, chronic exposure to these toxins does not provide mitochondria with sufficient time to repair and recalibrate, which might otherwise enable them to recover.

Although human studies demonstrate there may be no safe level for some industrial toxins [173], a non-linear dose–response is also observed, consistent with the activation of mitohormesis [166, 174]. Specifically, while low-dose exposures to certain toxins increase the risk of disease, the risk increase slows down, flattens out, or even decreases with increasing doses [169, 175]. Although these “challenging”, low-dose exposures often induce a degree of mitochondrial damage, this does not necessarily translate to the appearance of disease and can lead to benefits as mitochondria recover and adapt [166]. Importantly, this adaptive response is optimally activated by transient exposures and may be diminished by chronic exposure.

Interestingly, chronic exposure to near-zero doses of many industrial toxins may also lead to harmful effects on mitochondria [166]. This may be explained by the notion that chronic exposure to very low doses of toxins within a “sub-hormetic zone” may not sufficiently activate mitohormesis [169]. Even in the case of synthetic chemicals, an increased risk of disease has been associated with increasing doses within the sub-hormetic zone, but the risk flattens out as doses approach the hormetic zone. Again, a potentially important caveat to this observation is that some industrial toxins may be so highly toxic that no acceptable level exists [173]. Nonetheless, appreciable evidence indicates that attempting to maintain many industrial toxin exposures at near-zero levels may not be optimal for mitochondrial biology.

In the context of mitohormesis, unless a particular industrial toxin is proven to be highly toxic at any level, implementing public policies and personal behaviours aimed at balancing (1) transient, low-dose exposures (challenging, not excessive, stressor phases) with (2) prolonged, near-zero dose exposures (complete, not excessive, recovery phases) should be considered as a potential preventative and therapeutic strategy for neurodegenerative disorders.

### Dietary lifestyle

Arguably, the most profound changes in lifestyle over the last 50 years have occurred in the content and frequency of the human diet, which is characterized by a high intake of processed, carbohydrate-rich foods combined with multiple daily meals and snacks [9]. The modern dietary lifestyle represents less than 1% of the 10,000 or so years that most humans lived under an agrarian lifestyle, which itself has existed for less than 1% of the 2–3 million years that humans evolved under a hunter-gatherer lifestyle characterized by wild (pre-agrarian, unprocessed) foods and frequent periods of food scarcity [176]. Broadly speaking, the repeated consumption of processed, carbohydrate-rich foods constitutes a series of excessive stressor phases that lead to frequent blood glucose spikes, an overwhelming supply of nicotinamide adenine dinucleotide intermediates, and electron “overflow” along the mitochondrial ETC [177, 178]. In turn, electron overflow leads to excess ROS emission, a downward spiral of ROS-induced ROS release, and further damage [62], which culminates in mitochondrial fragmentation [50]. Simultaneously, a daily ritual of multiple meals and snacks leads to a series of insufficient recovery phases, given that the brief time intervals between meals do not provide mitochondria with adequate time to repair, recalibrate, and recover.

During feeding periods, the glucose spikes (and their sequelae) can be mitigated by low-carbohydrate diets, which restrict carbohydrates and increase fat to at least 40% of energy intake, and particularly by ketogenic diets, which restrict carbohydrates even further and increase fat to at least 70% of energy intake [179]. Restricting carbohydrate intake leads to a series of “challenging” nutritional stressor phases characterized by fewer and less severe blood glucose spikes, which mitigates electron overflow along the mitochondrial ETC and generates fewer ROS. A growing body of theoretical and clinical evidence also indicates that ketogenic diets can “rescue” brain and mitochondrial energy metabolism by generating ketones [180], a superior energy source for neurons that elicits fewer ROS, circumvents brain insulin resistance, and increases the expression of neurotrophic factors [181, 182]. Numerous animal studies show that ketogenic diets can benefit mitochondrial biology by restoring ion channel function, replenishing TCA cycle intermediates, enhancing respiration, and exerting pleiotropic neuroprotective effects, all of which would be expected to benefit people with neurodegenerative disorders [183]. Consistent with these findings, human interventional trials indicate that modified ketogenic diets can improve cognition, function, and quality of life in people with AD [184–187], as well as the motor and non-motor symptoms of PD [188–191]. Although the interventional evidence in people with ALS and HD is currently limited to case studies, these studies also hint at benefits [192, 193]. Beyond carbohydrate-restricted diets, the Mediterranean diet, which is typically based on unprocessed foods, may be beneficial for people with neurodegenerative disorders [194]. However, it is important to recognize that the vast majority of supportive evidence for the Mediterranean diet is derived from observational studies rather than interventional trials [195].

Outside the feeding periods, the incorporation of dedicated intermittent fasting periods (12–48 h) can relieve the chronic nutritional overload [196]. Fasting deprives the ETC of electrons, which leads to reduced ROS emission and an enhanced mitochondrial capacity to repair and recalibrate [182]. Fasting also induces many additional mechanisms that may be beneficial in neurodegenerative disorders, including the generation of ketones, increased expression of neurotrophic factors, and the stimulation of mitogenesis and mitophagy. Interventional studies in animal models show that fasting induces many beneficial metabolic changes and can slow the neurodegenerative process, leading to improved functional outcomes [197]. Benefits are also documented in animal models of monogenic disorders such as HD, including enhanced mitochondrial biology and clearance of the mutant HTT protein [198]. Despite these enticing findings, fasting-based interventional studies in people with neurodegenerative disorders are rare and currently relegated to case studies [192, 193].

In the context of mitohormesis, growing evidence suggests that an evolutionary hunter-gatherer dietary lifestyle aimed at balancing (1) minimally-processed, carbohydrate-restricted feeding periods (challenging, not excessive, stressor phases) with (2) intermittent fasting protocols (complete, not excessive, recovery phases) should be considered as a preventative and therapeutic strategy for neurodegenerative disorders.

### Cognitive, physical, and psychosocial behaviours

Significant changes in behaviour over the last 50 years have arisen in the setting of technological advances, which have undermined human exposure to a variety of authentic cognitive, physical, and psychosocial activities, as well as sleep and rest states. The human brain evolved over millions of years to learn, move, and interact within authentic environmental and social contexts, which cultivated positive psychosocial experiences associated with more abundant mitochondrial ETC proteins in the brain [199]. By contrast, the ease of living associated with technological advances in television, the internet, and digital media compromises the mitochondrial stressor phase. Simultaneously, chronic technology exposure disrupts natural sleep patterns and diverts bioenergetic resources away from natural resting states, which are essential for mitochondrial recovery [200, 201].

Preliminary evidence indicates that cognitive challenges can lead to physiological changes that may activate mitohormesis [168, 202]. Many studies indicate that environmental enrichment based on cognitive, sensory, and motor stimulation induces neuron remodelling and enhances cognitive and physical performance in animal models of AD, PD, ALS, and HD [203]. Interventional studies also show that cognitive stimulation leads to benefits in people with neurodegenerative disorders, which may be partially mediated by activated mitohormesis. A meta-analysis of 26 interventional studies found that multicomponent training, including lifestyle changes, enhances cognition in people with mild cognitive impairment [204]. A systematic review and meta-analysis of 15 interventional studies also discovered that playing board games preserves cognitive functions and brain structures in people with AD and dementia [205]. Furthermore, interventional studies demonstrate that multidisciplinary rehabilitation programs based on cognitive training, physical activities, and social events can lead to multiple cognitive benefits and reduce striatal and cortical atrophy in people with HD [206, 207].

Physical exercise can activate mitohormesis and improve many facets of mitochondrial biology [208]. In humans, even a single hour of challenging exercise induces significant ROS emission, oxidative damage, and mitochondrial impairments [209], including variable numbers of swollen mitochondria [210]. Following exercise, however, numerous cellular and mitochondrial adaptations occur, including anti-inflammatory, repair, and degradation processes [168, 211]. A wealth of interventional data indicates that exercise improves multiple outcome measures in people with neurodegenerative disorders, which may partly relate to the activation of mitohormesis. A systematic review of 28 randomized controlled trials found that intensive exercise performed over 2–3 days a week induces benefits in cognitive and physical function, functional independence, and neuropsychiatric symptoms in people with AD [212]. An updated systematic review of 33 randomized controlled trials demonstrated that intensive exercise leads to better functional capacity in PD [213]. Moreover, a systematic review of 7 randomized controlled trials showed that exercise enhances functional ability and pulmonary indices in ALS [214]. Furthermore, a review of 6 studies discovered that exercise induces benefits in cognition, motor symptoms, and mitochondrial content in HD [215]. Importantly, while insufficient exercise is a risk factor for many neurodegenerative disorders, excessive cardiovascular exercise (with insufficient recovery) can also lead to numerous adverse health outcomes [216], which may include an increased risk of developing ALS [113].

It has been suggested that mitochondria function as an intersection point within the body by sensing and translating psychosocial stressors into a range of biological modifications, including adaptive (or maladaptive) mitochondrial recalibrations [217]. Supportive evidence for this idea was provided by a recent systematic review of 23 animal studies, which showed that acute psychosocial stressors induce mitochondrial damage within hours, which in turn elicits a variety of protective mitochondrial recalibrations [218]. Conversely, chronic stress exposure facilitates maladaptive adjustments among mitochondria. Human observational studies also display an association between higher levels of social engagement and better late-life cognition [219], which may be partially mediated by activated mitohormesis. A systematic review of 39 observational and interventional studies found that a range of social activities, networks, supports, and relationships may improve cognitive function in older adults [220]. A smaller systematic review showed that social support interventions may reduce depression and benefit quality of life and self-esteem in AD [221].

Sleep has been described as a “mitorestorative” state that specifically protects and rejuvenates mitochondria [222]. Consistent with this hypothesis, sleep deprivation culminates in maladaptive metabolic and mitochondrial adjustments, including impaired neuronal ETC activity [223]. Sleep, by contrast, enhances the clearance of toxic metabolites from the brain, including ROS, as well as abnormal protein aggregates, such as amyloid-β [224]. Sleep also induces mitophagy, which keeps the mitochondrial pool healthy [225]. Human interventional studies show that better sleep leads to changes that would be expected to benefit people with neurodegenerative disorders, which may be partially mediated by enhanced mitochondrial recovery. A recent meta-analysis of 65 randomized controlled trials showed that improved sleep leads to better mental health, including improvements in depression, anxiety, rumination, stress, and psychosis [226].

Engaging in contemplative practices may facilitate a restorative state of “deep rest”, which channels energetic resources towards processes that optimize cellular function while diverting them away from energy-demanding stress states [201]. During a state of deep rest, energetic demands are low, which enables mitochondria to repair and recalibrate through mitogenesis and other restorative processes. A range of deep rest practices that would be expected to benefit people with neurodegenerative disorders may partly relate to enhanced mitochondrial recovery. A meta-analysis of 12 randomized controlled trials found that breathwork can be effective for mitigating stress and enhancing mental health [227]. A narrative review of 10 interventional studies also demonstrated that mindfulness or meditation may lead to less cognitive decline, a decrease in perceived stress, and positive changes in brain functional connectivity, volume, and blood flow in people with AD, dementia, or dementia-related memory conditions [228]. Moreover, a systematic review of 9 interventional studies discovered that mindfulness leads to cognitive benefits in PD [229].

In the context of mitohormesis, growing evidence indicates that an authentic lifestyle aimed at balancing (1) cognitive stimulation, exercise, and psychosocial engagement sessions (challenging, not excessive, stressor phases) with (2) dedicated sleep and deep rest practices (complete, not excessive, recovery phases) offers promise as a preventative and therapeutic strategy for neurodegenerative disorders. More research is required regarding their effectiveness.

## Conclusions

In keeping with Ovid’s statement that obvious effects often have hidden causes, we must carefully consider the cascade of events that underlie neurodegenerative disorders. The longstanding split of these disorders based on their hallmark clinical, anatomical, and pathological features leads to the perception that they are focal neurological disorders amenable to targeted, suppressive treatments. Splitting is useful for diagnosis and management. However, treatments based on splitting alone have struggled to produce clinically meaningful outcomes. Using an alternative scientific framework, neurodegenerative disorders can be lumped by a shared feature of impaired mitochondrial biology. Lumping more accurately depicts these disorders as multisystemic disorders in need of multisystemic, restorative therapies. Since lumping emphasizes hidden causes rather than visible effects, it may get us further along the path of recognizing the modifiable etiological factors of neurodegeneration that are crucial for guiding prevention and therapy.

In this article, we have presented a conceptualization of neurodegenerative disorders as metabolic icebergs, which conveys the hallmark clinical, anatomical, and pathological features (tip) as late-stage effects of impaired mitochondrial biology (bulk), which in turn is induced by a suite of environmental and genetic factors (base). Mitohormesis-activating strategies can be sustainably harnessed to facilitate a balanced oscillation between mitochondrial stressor and recovery phases, leading to the repair, recalibration, and recovery of mitochondria throughout the body. Ultimately, we may find success in treating the rising tide of neurodegenerative disorders by addressing the hidden energetic and metabolic causes beneath the clinical waterline, rather than by focusing exclusively on the visible effects that naturally emerge, in time, above it.

## Data Availability

Not applicable.

## Abbreviations

AD: Alzheimer’s disease
PD: Parkinson’s disease
ALS: Amyotrophic lateral sclerosis
HD: Huntington’s disease
ATP: Adenosine triphosphate
ROS: Reactive oxygen species
TCA: Tricarboxylic acid
ETC: Electron transport chain
mtDNA: Mitochondrial DNA
PET: Positron emission tomography
APOE4: Apolipoprotein E4
PRKN: Parkin
PINK1: Phosphate and tensin homolog-induced kinase 1
PGC-1α: Peroxisome proliferator-activated receptor-γ coactivator-1α
C9orf72: Chromosome 9 open reading frame 72
SOD1: Superoxide dismutase 1
FUS: Fused in sarcoma
TDP-43: Transactive response DNA binding protein 43
HTT: Huntingtin
